# Lack of Privileged Access to Awareness for Rewarding Social Scenes in Autism Spectrum Disorder

**DOI:** 10.1007/s10803-018-3595-9

**Published:** 2018-05-04

**Authors:** Katie L. H. Gray, Anthony Haffey, Hristina L. Mihaylova, Bhismadev Chakrabarti

**Affiliations:** Centre for Autism, School of Psychology and Clinical Language Sciences, University of Reading, Reading, RG6 6AL UK

**Keywords:** Awareness, Social motivation, Reward, Autism

## Abstract

Reduced social motivation is hypothesised to underlie social behavioural symptoms of Autism Spectrum Disorder (ASD). The extent to which rewarding social stimuli are granted privileged access to awareness in ASD is currently unknown. We use continuous flash suppression to investigate whether individuals with and without ASD show privileged access to awareness for social over nonsocial rewarding scenes that are closely matched for stimulus features. Strong evidence for a privileged access to awareness for rewarding social over nonsocial scenes was observed in neurotypical adults. No such privileged access was seen in ASD individuals, and moderate support for the null model was noted. These results suggest that the purported deficits in social motivation in ASD may extend to early processing mechanisms.

## Introduction

Given the brain’s capacity limitations, incoming sensory information must be selected for further processing. Social stimuli, such as faces and bodies, attract attention (Bindemann et al. 2005; Crouzet et al. 2010; Downing et al. 2004), suggesting that they are prioritised in the competition for selection. When presented in simple arrays, social images attract attention more quickly than other objects, even in young infants (Gluckman and Johnson 2013). However, we rarely view social information in isolation; it is more typically processed alongside surrounding contextual information. When presented in complex visual scenes, social information has been found to capture attention rapidly in typical observers (Fletcher-Watson et al. 2008; Birmingham et al. 2008a, b, 2009). Using eye-movement measures, the bias towards social information in natural images has been found as early as the first saccade (Fletcher-Watson et al. 2008), despite it not being the most visually salient information in the scene (Birmingham et al. 2009).

The extent to which social stimuli capture attention may depend on characteristics of the observer. Individuals with Autism Spectrum Disorders (ASD) display characteristic abnormalities in their social interactions and communication (American Psychiatric Association 2013). There is evidence to suggest that rapid orienting to social stimuli is disrupted in ASD (Dawson et al. 1998; Klin et al. 2009). The social motivation theory of autism suggests that social stimuli elicit lower levels of reward in ASD than in typically developing individuals (Chevallier et al. 2012). For example, using an incentive delay task, Demurie et al. (2011) found that children with ASD responded faster to monetary than social rewards, whereas typically developing children responded equally quickly to both reward types. Adults with ASD have been reported to be slower in orienting to social stimuli when presented within an array containing nonsocial stimuli (Wang et al. 2014), though this effect is particularly strong when the competing nonsocial stimuli pertain to the circumscribed interests commonly noted in ASD (Sasson and Touchstone 2014). Notably, the evidence discussed above has presented stimuli at or above the threshold of awareness.

In typical observers, the rapid orienting response to social stimuli may occur early in the visual processing stream. Faces and bodies are not only granted prioritised access to attention, they are also granted prioritised access to visual awareness (Stein et al. 2012). To measure access to visual awareness, continuous flash suppression (CFS) has been commonly used, in which an image presented to one eye is suppressed for a prolonged period of time by a dynamic mask presented to the other eye (Tsuchiya and Koch 2005). The time it takes for a stimulus to enter awareness (or *break* suppression) is measured, and is thought to index stimulus salience (Stein et al. 2011a). For example, preferential selection of threat-related information has been suggested to hold an evolutionary advantage, and indeed, fearful faces break suppression more quickly than neutral faces (Gray et al. 2013; Hedger et al. 2016). Despite upright faces emerging from suppression more quickly than inverted faces (Gray et al. 2013), and faces emerging more quickly than objects (Stein et al. 2012), to date, no study has tested whether rewarding social information is granted privileged access to awareness when presented in complex natural scenes.

Recently, it has been found that individuals with ASD display typical prioritisation of highly simplified ‘protoface’ stimuli, in both attention (using an attentional-cueing paradigm; Shah et al. 2013) and visual awareness (using CFS; Akechi et al. 2015). However, using more natural face images in CFS, Akechi et al. (2014) found non-typical responses to eye-gaze in individuals with ASD. Typically developing adolescents showed a standard eye-gaze effect (Stein et al. 2011b), whereby direct-gaze faces broke suppression more quickly than averted-gaze faces. However, adolescents with ASD did not show a direct-gaze preference in CFS, suggesting the mechanism for deficits in eye-gaze detection in ASD occur early in the visual processing stream (Akechi et al. 2014).

Here, we ask whether rewarding social scenes are granted privileged access to awareness when compared to non-social scenes. We presented positively valenced visual scenes outside of conscious awareness using CFS and measured, using response times, how quickly the scenes emerged from suppression. In Experiment 1, we addressed this question in a sample of typical adults; in Experiment 2, we tested individuals with ASD. Given the inherent difficulty in matching samples effectively (Blackford 2006; Facon et al. 2011), and the known problems in statistical covariation methods (Miller and Chapman 2001), we have reported these samples over two identical experiments. We have complemented traditional null significance hypothesis testing (NHST) with corresponding Bayesian analyses, which allowed us to quantify the evidence for an absence of an effect (Rouder et al. 2012).

## Experiment 1

### Methods

#### Participants

Thirty-eight naïve adults (Mean age = 21.16; *SD* = 3.77; 11 males) with normal or corrected-to-normal visual acuity participated in Experiment 1. For both experiments, ethical clearance was granted by the University of Reading research ethics committee, and all participants gave informed consent.

#### Materials

**Fig. 1:**
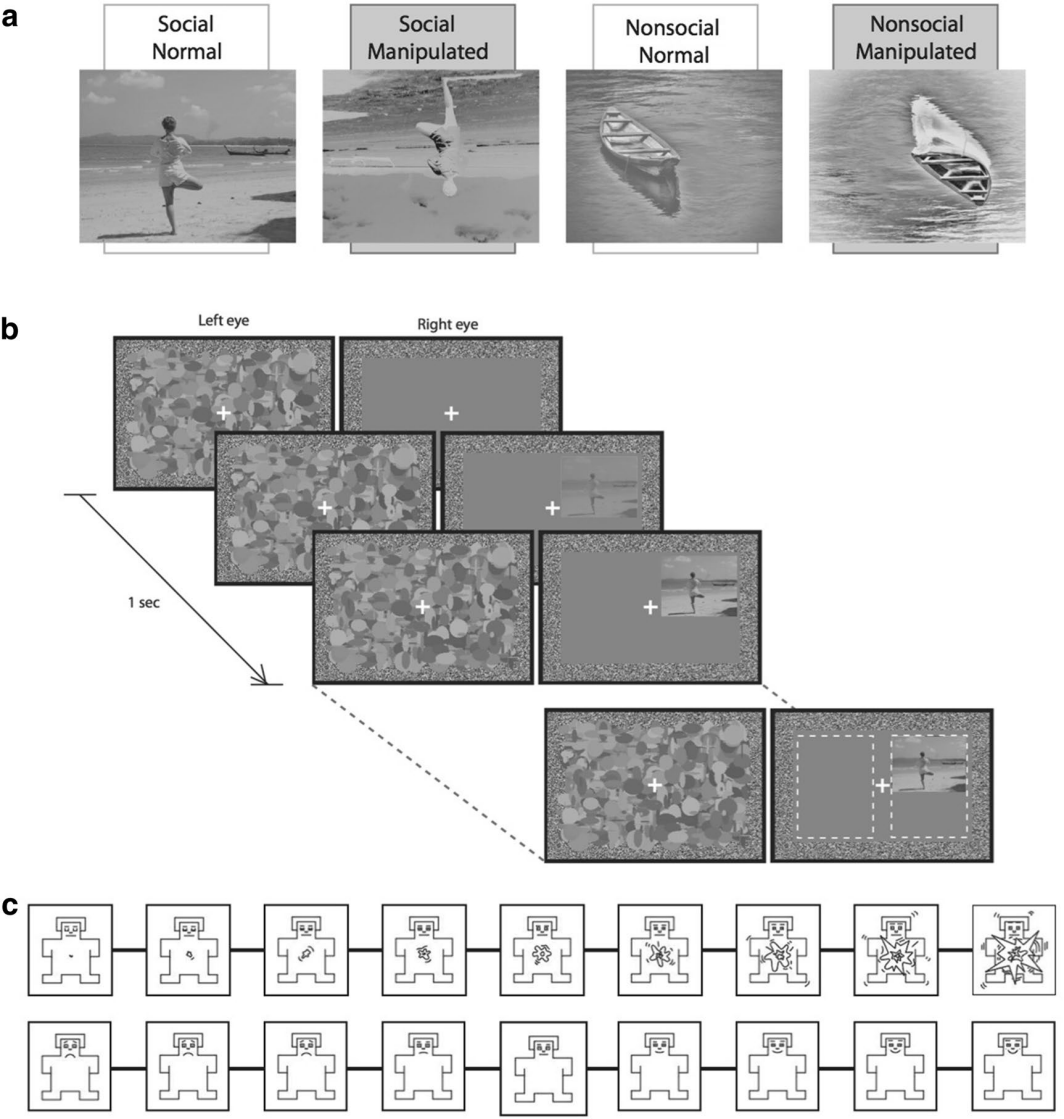
**a** Complex visual scenes containing social information, or nonsocial information were presented normally, or manipulated (with negative contrast and spatially inverted). Example images (not used in the study) were taken from OASIS (Kurdi et al. 2017). **b** Trial schematic. The target was presented to one eye, and random dynamic noise to the other. Participants selected the target location (left or right of fixation). **c** Arousal (top) and valence (bottom) ratings were taken for each image using Self Assessment Manikins (Bradley and Lang 1994)

The 16 images used were taken from the International Affective Picture System (IAPS; Lang et al. 2008) and publicly available images from the internet (free for non-commercial reuse). Images were selected based on their valence and arousal ratings from previous studies on neurotypical university students of both sexes (N = 145). Both social images (*M* = 6.39, *SD* = .46) and non-social images (*M* = 6.32, *SD* = .43) were rated similarly positive, and arousal ratings were comparable for social images (*M* = 4.35, *SD* = .71) and non-social images (*M* = 4.10, *SD* = .43). All images also had canonical orientation, were presented in grey-scale, and were matched on mean luminance and root-mean-square contrast. The scenes were prepared and presented using MATLAB (Mathworks) with PsychToolbox extensions (Brainard 1997; Pelli 1997). Local low-level stimulus properties were controlled for using an additional condition in which the images were presented with negated contrast, and spatial inversion (Fig. 1a). These manipulations reduce face recognition accuracy (Galper 1970; Yin 1969), and they also delay (Torralba and Sinha 2001) and increase thresholds (Liu-Shuang et al. 2015) for face detection. They are also thought to disrupt recognition of complex scenes; performance on matching tasks is significantly disrupted by scene inversion (Epstein et al. 2006), and the combination of negated contrast and spatial inversion has been found to significantly reduce face detection accuracy when faces are presented within a visual scene context (Torralba and Sinha 2001).

#### Procedure

Images of scenes (2.6° × 3.5° of visual angle at a viewing distance of 60 cm) were presented under interocular suppression using a mirror stereoscope. On each trial, a scene was presented to one eye, and a high-contrast mask pattern updating at 10 times/s was presented to the other eye (Fig. 1b). The contrast of the image was increased linearly over the first second to reduce the likelihood of break-through due to onset transients; the images then remained at full contrast until the observer responded.

The position of the image was randomly assigned to the left or right of the visual field, and the position vertically within the visual field was randomly jittered on each trial. A binocularly viewed frame and fixation cross were used to facilitate convergence. Participants were required to indicate, using the arrow keys, which side of fixation they ‘detected anything other than noise’. They were also required to fixate on the fixation cross for the duration of the trial. Head movements were controlled using a chin rest and forehead bar. Each participant completed six practice trials before completing the 256 trials of the experiment (two social content (social, nonsocial) × two stimulus manipulations (normal, manipulated) × eight images × eight repetitions), balanced across presentation location and eye.

Following the CFS experiment, participants rated the normal stimuli for valence and arousal. Each stimulus was presented twice—once with a nine point valence scale presented beneath it, and again with a nine point arousal scale. The scales were represented by manikins, which participants clicked on to convey their response (Bradley and Lang 1994; Fig. 1c). If participants failed to respond within 6 s the trial proceeded without a response.

### Data Analysis

Mean response times were calculated from correct trials. Four individuals were excluded as they were at chance level on the location task. For the remaining participants, the number of incorrect trials were small (*M* = 3.46%), and did not differ over presentation conditions [*F*(3,99) = 2.28, *p* > .05]. Response times ± 3SDs from the mean for each observer were removed from further analysis (mean number of trials excluded = .57%) and did not differ over presentation conditions [*F*(3,99) = 2.36, *p* > .05]. Bayes factors were computed using JASP (Love et al. 2015) with default prior width; we report evidence that the data were more likely under the alternative model compared to the null model, and interpreted Bayes factors (BF) of < 3 as anecdotal, 3–10 as substantial, 10–30 as strong, 30–100 as very strong, and > 100 as decisive evidence (Jeffereys 1961). In all Bayesian analyses reported, we included subject, age and gender as random factors.

## Results and Discussion

Our sample rated social (valence: *M* = 6.29, *SD* = 1.18; arousal: *M* = 4.88, *SD* = .72) and non-social (valence: *M* = 5.83, *SD* = .6; arousal: *M* = 4.92, *SD* = .76) scenes similarly for both valence [*t*(14) = .96, *p* = .35], and arousal [*t*(14) = .10, *p* = .92]. This is unsurprising, given that the images were selected based on their similarity in these dimensions.

**Fig. 2:**
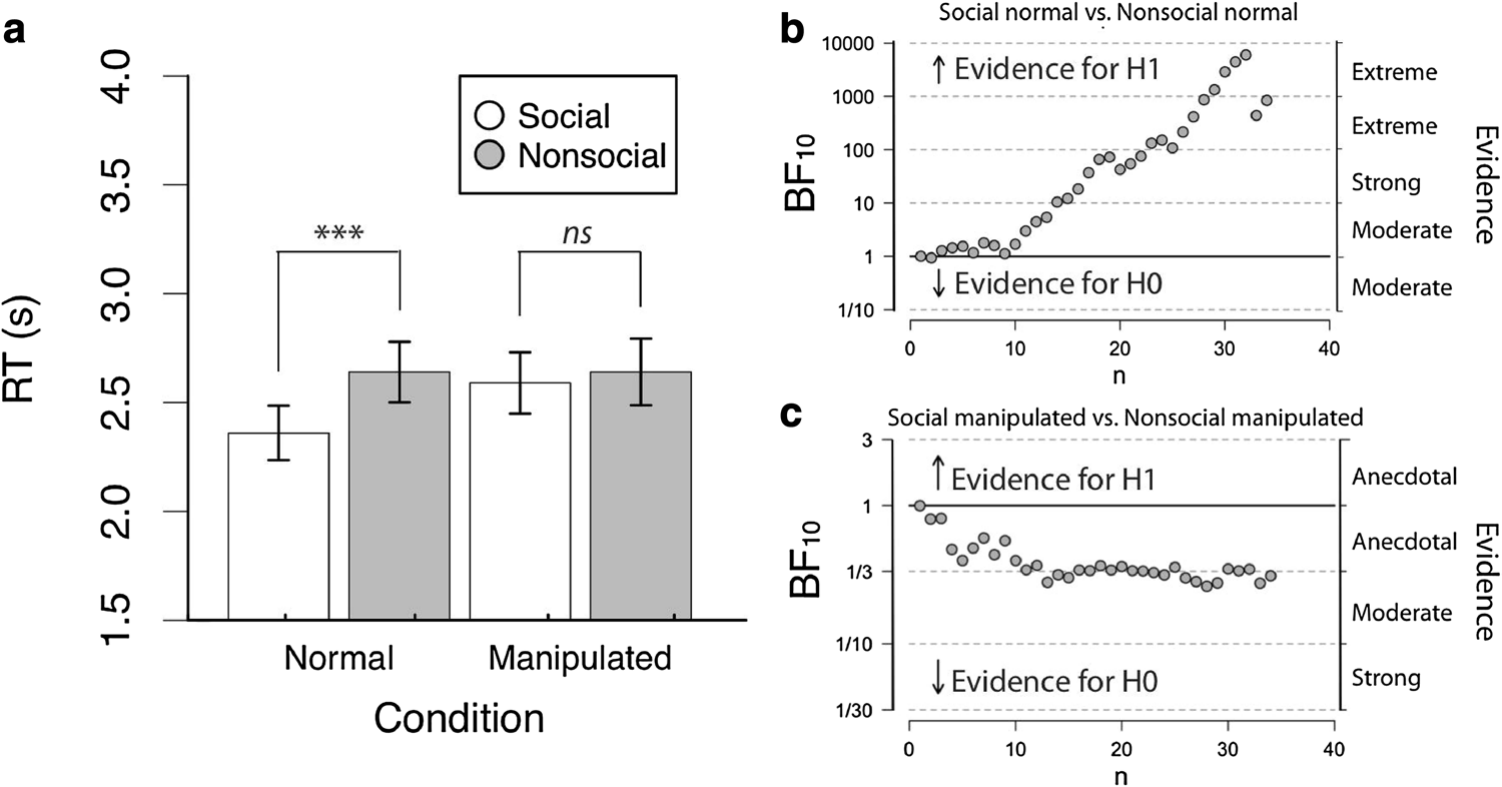
Results from Experiment 1 (typical observers) analysed using: **a** null significance hypothesis testing, where *** denotes *p* < .001, ** denotes *p* < .01, * denotes *p* < .05. Error bars denote ± 1SEM; and sequential analysis from Bayesian within participant *t*-tests for **b** social normal versus nonsocial normal and **c** social manipulated versus nonsocial manipulated comparisons

A 2 × 2 repeated measures ANOVA on the mean response times revealed a main effect of Social Content [*F*(1,33) = 12.96, *p* = .001, ${\eta}_{p}^{2}=.28$], whereby social stimuli were responded to more quickly than non-social stimuli. There was also a main effect of Stimulus Manipulation [*F*(1,33) = 4.78, *p* < .05, ${\eta}_{p}^{2}=.13$], whereby normal stimuli were responded to more quickly than manipulated stimuli. These main effects were qualified by a significant interaction between Social Content and Stimulus Manipulation [*F*(1,33) = 16.41, *p* < .001, ${\eta}_{p}^{2}=.33$]. Pairwise contrasts revealed evidence for preferential access to awareness for social stimuli (social normal: *M* = 2.36, *SD* = .73; non-social normal, *M* = 2.64, *SD* = .81; *t*(33) = 4.87, *p* < .001), but only when they were not manipulated (social manipulated: *M* = 2.59, *SD* = .82; non-social manipulated: *M* = 2.64, *SD* = .89; *t*(33) = 1.06, *p* = .29; see Fig. 2a).

The results of the NHST ANOVA were complemented by a Bayes factor ANOVA (Rouder et al. 2012), which revealed evidence that each main effect model was preferred to the null model (Social Content: BF = 47.18; Stimulus Manipulation: BF = 2.79). However, the interaction model (BF = 711.71) was strongly preferred over the combined main effects model (BF = 187.67), concurring with the repeated-measures NHST ANOVA and suggesting that Social Content and Stimulus Manipulation interact in their effect on response times (see Fig. 2b, c).

## Experiment 2

Results from Experiment 1 suggest that social scenes have privileged access to visual awareness. Given that simple social stimuli appear to access awareness normally in individuals with ASD (Akechi et al. 2015), but complex social stimuli do not (Akechi et al. 2014), we next explored the extent to which rewarding social scenes are granted privileged access to awareness in a sample of individuals with ASD.

### Method

#### Participants

Thirty naïve adults (Mean age = 37.19; *SD* = 13.73; 14 males) with a DSM-IV TR based diagnosis of ASD from a recognised clinic were recruited through the Berkshire Autism Research Network database of research volunteers. As an additional check on diagnostic status, all participants filled in the Autism Spectrum Quotient (AQ). All participants but one scored equal to or above the suggested diagnostic cut-off score of 26 on the AQ (Woodbury-Smith et al. 2005). All participants were also assessed with the Autism Diagnostic Observation Schedule (ADOS; Lord et al. 1999). Twenty of the participants met criteria for ASD according to the ADOS, while eight did not.^1^ Two individuals withdrew from the ADOS as they found the process upsetting. To capture cognitive ability, 23 of the participants completed the Raven’s progressive matrices (Raven 1998). The remaining seven were not tested due to an administrative error. This task estimates the participant’s cognitive ability as a percentile of the general population. The average percentile of these participants was 54.04 (*SD* = 28.66), suggesting that cognitive functioning of these participants as a group is comparable to neurotypical participants. Prior to testing, participants’ ability to binocularly fuse visual stimuli was tested using the RanDot graded circles test (Stereo Optical Company), which tests fine depth discrimination at different levels of stereopsis (400–20 s of arc). Three participants were excluded, as they did not reach the minimum threshold (400 s of arc). All other participants reached a minimum threshold of 70 s of arc.

#### Materials and Procedure

The stimuli and procedure were the same as Experiment 1.

### Data Analysis

Mean response times were again calculated from correct trials. The number of incorrect trials were small (*M* = 3.82%), and did not differ over presentation conditions [*F*(2.1, 54.6) = 2.36, *p* > .1]. Response times ± 3SDs from the mean for each observer were removed from further analysis (mean number of trials excluded = 2.14%) and did not differ over presentation conditions [*F*(3,78) = .73, *p* > .5].

### Results and Discussion

Our ASD sample rated social (valence: *M* = 5.78, *SD* = .64; arousal: *M* = 4.93, *SD* = .90) and non-social (valence: *M* = 6.13, *SD* = .75; arousal: *M* = 4.55, *SD* = .35) scenes similarly for both valence [*t*(14) = 1.03, *p* = .32], and arousal [*t*(14) = 1.12, *p* = .28].

**Fig. 3:**
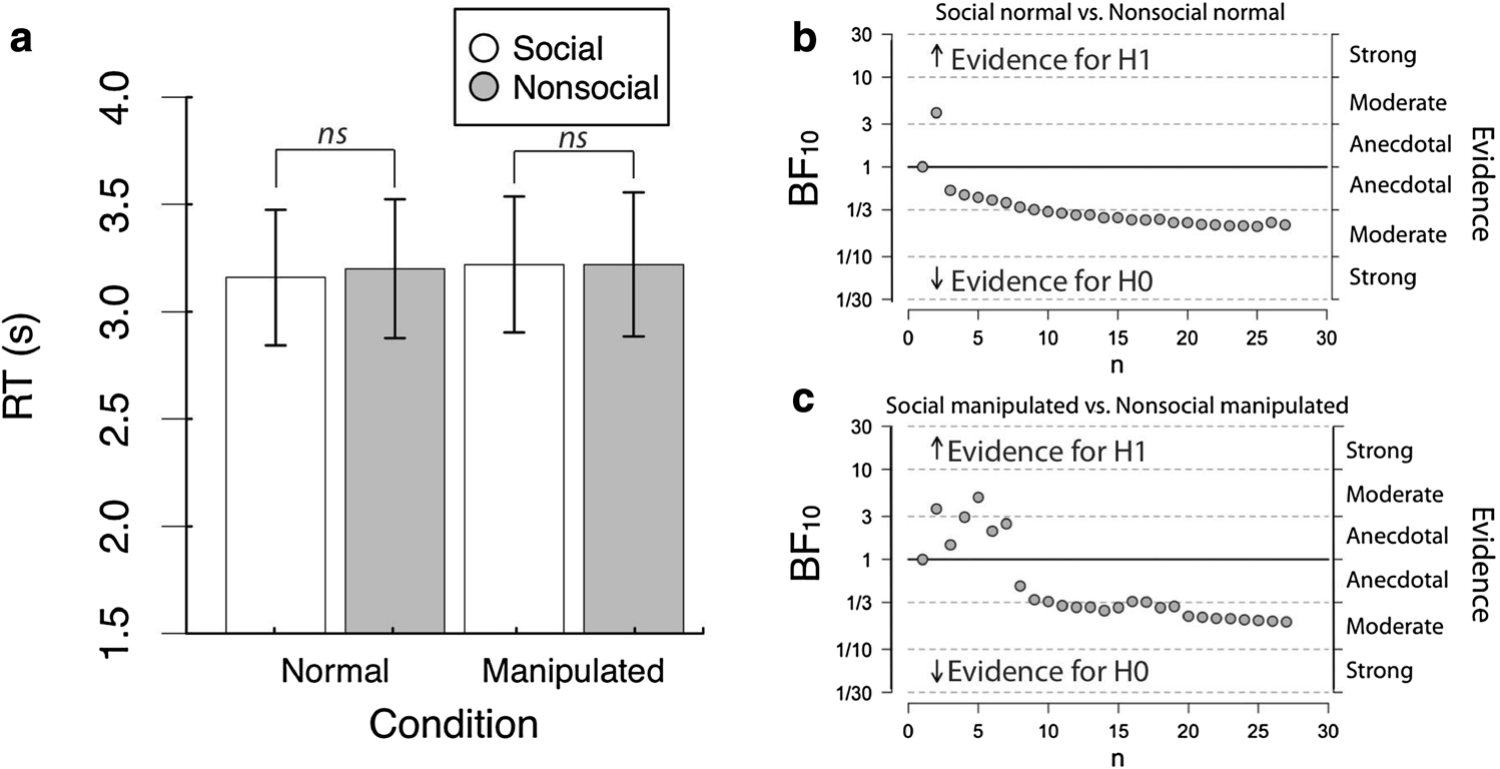
Results from Experiment 2 (ASD observers) analysed using: **a** null significance hypothesis testing, where *** denotes *p* < .001, ** denotes *p* < .01, * denotes *p* < .05. Error bars denote ± 1SEM; and sequential analysis from Bayesian t-tests for **b** social normal versus nonsocial normal and **c** social manipulated versus nonsocial manipulated comparisons

A 2 × 2 repeated measures ANOVA on the mean response times revealed no main effect of Social Content [*F*(1,26) = .06, *p* = .81, ${\eta}_{p}^{2}=.002$], nor of Stimulus Manipulation [*F*(1,26) = .24, *p* = .63, ${\eta}_{p}^{2}=.009$], and no interaction between Social Content and Stimulus Manipulation [*F*(1,26) = .07, *p* = .79, ${\eta}_{p}^{2}=.003$] (Fig. 3a).

The results of the ANOVA were complemented by a Bayes factor ANOVA (Rouder et al. 2012) with default prior scales, which revealed evidence for the null model was strongly preferred to the main effects model (BF = .046) and the interaction model (BF = .013). This concurs with the standard (NHST) repeated-measures ANOVA, and suggests strong evidence for the null model (see Fig. 3b,c).

## General Discussion

In typical observers, social stimuli were found to break suppression more quickly than nonsocial stimuli; however, when manipulated (inverted and contrast negated), both stimulus types were equally slow to break suppression. Using the same experimental design, procedure, and setup, we then tested individuals with a diagnosis of ASD. In both the normal, and manipulated conditions, we found no effect of social content on time to break suppression. These results provide evidence that social stimuli enjoy privileged access to awareness over non-social images in typical observers, but this is not the case in observers with ASD.

The lack of a preference for social information in individuals with ASD was not due to participants having difficulties fusing the stimuli in the stereoscope, as all included participants had good stereoscopic depth perception. As all other methodological details were the same as Experiment 1, these results suggest that individuals with ASD have a deficit in processing social stimuli at an early stage of the visual processing hierarchy. It has been suggested that individuals with ASD process ‘protofaces’ (basic face-like stimuli) in a similar way to typical participants (Shah et al. 2013; Akechi et al. 2015). However, using photographic images, no eye-gaze effect was reported in individuals with ASD (Akechi et al. 2014). Our findings also suggest that the processing of complex rewarding social information outside of conscious awareness is aberrant in ASD.

Social reward signals play an important role from early development in facilitating learning (Wu et al. 2011). Lack of privileged access to these signals can arguably have an adverse effect on social learning, which in turn can lead to social behavioural difficulties often reported by individuals with ASD. A large number of studies have reported atypical processing of social stimuli in individuals with ASD (Chita-Tegmark 2016; Scott-Van Zeeland et al. 2010; Izuma et al. 2008; Kohls et al. 2012; Richey et al. 2013; Delmonte et al. 2012). A subset of these studies do not find any evidence for differential processing of social vs. nonsocial stimuli in individuals with ASD (Chita-Tegmark 2016). Importantly however, none of these studies have presented stimuli below the threshold of awareness, and thus the current paradigm provides a novel perspective to this set of observations on reduced preferential processing of social stimuli in ASD.

The ASD sample had longer/slower response times during interocular suppression than our sample in Experiment 1. However, the null effects in Experiment 2 are unlikely due to domain-general processes, such as slowed visual mechanisms or slowed motoric speed, as these factors should affect all stimulus categories equally. We discuss two possibilities below. First, if motoric slowness in the ASD group was responsible for the group difference, then one should expect the same pattern of social advantage in ASD participants as noted in Experiment 1, but at longer RTs for all conditions. We do not see such a pattern, and hence the evidence for this possibility is weak. Second, it is possible that the slowed motoric speed causes a ceiling effect, which can arise if the magnitude of the social advantage effect is an order of magnitude smaller than the extent of motoric slowness in the ASD group. This second possibility also appears unlikely since the social advantage effect (~ 0.2 s) is not an order of magnitude smaller than the extent motoric slowness in the ASD group (~ 0.6 s). In light of these observations, we suggest that the elevated RTs for the ASD sample relative to the sample in Experiment 1 is likely due to domain general processes associated with age.

The sample of ASD participants in the present experiment included, by chance, similar number of males and females. ASD tends to occur more frequently in males (Baron-Cohen et al. 2011), and there is evidence to suggest that social processing in ASD somewhat depends on participants’ sex (Coffman et al. 2015). Although underpowered to explore in current experiment, sex differences in privileged processing of social prioritisation may be a fruitful line of research in the future. As the participants in the current study were high-functioning, our results may not be generalizable to the ASD population as a whole. While all ASD participants had a verifiable clinical diagnosis using DSM criteria, some of them did not meet the cut-off score on the ADOS. This is not an uncommon observation, as the ADOS was neither developed nor optimised for diagnosing high-functioning adults with ASD, such as the participants in the current study. However, in order to ensure that none of the reported results are driven by the eight individuals who did not meet the cut-off score on ADOS, all analyses were rerun including only participants who did so. This re-analysis revealed no significant difference in the results from those reported on the full sample.

Besides the implications of the current results for ASD, these experiments provide new insights into the processing of complex social scenes in neurotypicals. Given that previous studies have found upright faces emerge from suppression more quickly than inverted faces (Gray et al. 2013), and objects (Stein et al. 2012), it is unsurprising that social stimuli emerge more quickly than non-social stimuli. Previous findings have shown that complex visual scenes containing threat-related information are not preferentially processed over neutral scenes in CFS (Hedger et al. 2015). Using positively valenced visual scenes, ours is the first study to show the prioritisation of social content in CFS. Social interactions often involve responding to very quick or subtle cues, and having privileged access to such cues can potentially result in more efficient planning of responses.

In conclusion, these two experiments demonstrate an advantage for rewarding social stimuli to receive prioritised visual processing compared to similarly valenced nonsocial stimuli. This difference is not explained by stimulus features, as inverted and negated versions of these stimuli do not show this observed advantage. Crucially, no such advantage for social stimuli is seen in individuals with ASD. These results are informative for the social motivation model of understanding the ASD phenotype, by providing fresh evidence on the processing of social stimuli from early visual mechanisms.

## Footnotes

1 Results were near identical when these participants (or the participant not exceeding cutoff on the AQ) were excluded from the analysis.
